# An investigation of professionalism reflected by student comments on formative virtual patient encounters

**DOI:** 10.1186/s12909-016-0840-9

**Published:** 2017-01-06

**Authors:** Ting Dong, William Kelly, Meredith Hays, Norman B. Berman, Steven J. Durning

**Affiliations:** 1 Uniformed Services University of the Health Sciences (USUHS), Bethesda, USA; 2 Geisel School of Medicine at Dartmouth, 1 Rope Ferry Road, Hanover, NH 03755 USA; 3 Department of Medicine (A3068), USUHS, 4301 Jones Bridge Road, Bethesda, MD 20814 USA

**Keywords:** Virtual patient encounter, Professionalism, Residency evaluation

## Abstract

**Background:**

This study explored the use of virtual patient generated data by investigating the association between students’ unprofessional patient summary statements, which they entered during an on-line virtual patient case, and detection of their future unprofessional behavior.

**Method:**

At the USUHS, students complete a number of virtual patient encounters, including a patient summary, to meet the clerkship requirements of Internal Medicine, Family Medicine, and Pediatrics. We reviewed the summary statements of 343 students who graduated in 2012 and 2013. Each statement was rated with regard to four features: *Unprofessional*, *Professional*, *Equivocal* (could be construed as unprofessional), and *Unanswered* (students did not enter a statement). We also combined *Unprofessional* and *Equivocal* into a new category to indicate a statement receiving either rating. We then examined the associations of students’ scores on these categories (i.e. whether received a particular rating or not) and Expertise score and Professionalism score reflected by a post-graduate year one (PGY-1) program director (PD) evaluation form. The PD forms contained 58 Likert-scale items designed to measure the two constructs (Expertise and Professionalism).

**Results:**

The inter-rater reliability of statements coding was high (Cohen’s Kappa = .97). The measure of receiving an *Unprofessional* or *Equivocal* rating was significantly correlated with lower Expertise score (*r* = −.19, *P* < .05) as well as lower Professionalism score (*r* = −.17, *P* < .05) during PGY-1.

**Conclusion:**

Incident reports and review of routine student evaluations are what most schools rely on to identify the majority of professionalism lapses. Unprofessionalism reflected in student entries may provide additional markers foreshadowing subsequent unprofessional behavior.

## Background

Professionalism is a required competency for medical students, residents, and practicing physicians [1]. Many medical specialty organizations highlight the critical nature of professionalism in their charters. From these various organizations, principles inherent to professionalism are honor, integrity, respect and accountability of actions [2]. Despite this growing understanding of professionalism, the literature is replete with unprofessional behavior [1, 3]. Prior work has shown the concerning trend that unprofessional behavior in medical school is associated with increased likelihood of public reprimand and state medical board licenses being revoked, typically 20 or more years from graduation [2].

One way to address concerns regarding unprofessional behavior is enhancing our ability to detect signs of and potentially remediate (or make decisions for disenrollment) such behavior early. A number of measures to detect unprofessionalism have been investigated. These have included tracking individuals who miss required administrative tasks such as completing immunizations [4], and concerning comments from recommendation letters [2] or from faculty evaluators [5, 6]. Such findings about early unprofessional behavior have been shown associated with formal or graded evaluations (“higher stakes” evaluations), albeit small effect sizes reported in some of the studies.

In recent years, it is shown that Internet posting of unprofessional content is common among medical students and residents [7]. The online environment provides a previously unavailable window into students’ actions and activities around their learning. Meanwhile, researchers suggest the use of virtual patients to address challenges facing medical education, especially in clinical clerkship education [8]. The goal of the current study was to explore the association between investigator rated professionalism of student comments during clerkship rotations using a virtual patient platform known as MedU with future performance. We investigated whether answers on ungraded or other formative, “lower stakes” assignments are associated with future performance.

MedU is currently used at many medical schools [9]. These virtual patient encounters are completed during many clerkship rotations and are designed for learning and feedback. Virtual patient encounters are interactive patient scenarios that are designed to develop a student’s medical knowledge and clinical reasoning. Students work through a portion of the case and are then asked to provide a brief free text summary of the encounter as if presenting the patient to their internal medicine clerkship preceptor.

At the Uniformed Services University of the Health Sciences (USUHS), like many other medical schools, students are required to complete a number of virtual patient encounters to meet this clerkship requirement. At the time of this study, the students received no feedback from the computer system or from their preceptor related to the content of the submitted summary statement; students are given full credit with merely completing the cases for this “low stakes” assignment.

Review of a random sample of student free text summary statements by several authors (WK, SJD), revealed that some students’ summaries were unequivocally unprofessional. We therefore decided to investigate this phenomenon further for purposes of determining if such a measure could help improve our early detection of unprofessional behavior. More specifically, we hypothesized that unprofessional summary statements would be associated with future unprofessionalism, measured by the professionalism factor contained in the students’ first-year residency evaluation completed by their program directors, for which we have previously gathered reliability and validity evidence [5, 6]. We also examined the associations between this early professionalism measure and common academic performance indicators such as GPA, USMLE Step 1 score, and Step 2 clinical knowledge (CK) score.

## Method

### Study context and participants

This study was conducted by MedU and the Long-Term Career Outcome Study (LTCOS) at the F. Edward Hébert School of Medicine, USUHS. As the United States’ only federal medical school, USUHS matriculates approximately 170 medical students annually and, at the time of this study, offered a traditional four-year curriculum: two years of basic science courses followed by two years of clinical rotations (clerkships). The purpose of LTCOS is to collect and analyze a variety of quantitative and qualitative data before, during, and after medical school so that USUHS can more effectively evaluate the success of its graduates and educational programs. The participants were USUHS medical students who graduated in 2012 and 2013 (*n* = 343).

### Study procedures

Students completed MedU virtual patients (http://www.med-u.org) during three of our core clerkships: internal medicine, pediatrics and during family medicine. The expectations were provided to students during both student clerkship mandatory orientations as well as in the clerkship handbook – “You may accomplish these five (5) SIMPLE cases at any time during the 15 week clerkship block, although we recommend you do one each week during the psychiatry clerkship. MedU gives us a report of completion (and time spent) on each case. Failure to complete the five (5) SIMPLE cases requirement may result in Department of Medicine Education Committee (DOMEC) review”. The students were told at other times that answers were centrally monitored.

Investigators reviewed the summary statements of all students during the study period and coded their statements based on our rubric described below. We initially devised a coding rubric that sought to classify summary statements as unprofessional, neutral, or professional. A sample (approximately 10%) of comments were coded and discussed between the four coders (SJD, WK, MH). We subsequently refined our coding scheme, resolving disagreements, and included another category for unanswered summary statements. Each summary statement was then reviewed and coded by at least two raters as: Unprofessional (comment rated unprofessional), Professional (comment not rated unprofessional), Equivocal (could be construed as unprofessional) or Unanswered (left blank). Following subsequent coding of all the comments in our sample (100%) inter-rater reliability was high (Cohen’s Kappa = .97). All disagreements were resolved by consensus.

### Measures

To answer the research questions, we investigated the associations among the four possible codes with the following measures: medical school cumulative GPA, United States Medical Licensing Examination (USMLE) Step 1 score, Step 2 clinical knowledge (CK) score, appearance before the department of medicine’s clinical competency committee (DOMEC), which is a marker for a struggling student (explained below), and Expertise score and Professionalism score of post-graduate year 1 (PGY-1) program director (PD) evaluations.

The USMLE is a single program consisting of four separate examinations designed to assess an examinee’s understanding of and ability to apply concepts and principles that are important in health, disease, and effective patient care. Students in this sample completed Step 1, which focuses on understanding of basic sciences, after their first two years of medical school. Students completed Step 2 (CK and CS) during their fourth year of medical school. Step 2 CK is more clinically oriented compared with Step 1. During the students’ 12-week internal medicine clerkship, each of the teaching faculty make determinations on each student using the Reporter-Interpreter-Manager-Educator (RIME) framework [10, 11]. The RIME framework is a step-wise progression of professional growth during medical school. Reporters can gather and report clinical information and distinguish between important and unimportant information. Those at the Interpreter level should be able to identify and prioritize problems as well as develop a differential diagnosis. Managers should be able to develop and defend a plan based on the patients’ problems. Finally, an Educator can do all of the previous as well as participate in the education of the rest of the team [10]. This is converted to numerical points, weighted based on time spent with the student, and added to written examination score points to come up with a grade. Any student who has “less than Reporter” or other concerning comments from any teachers or fails the National Board of Medical Examiners (NBME) subject examination undergoes clinical competency committee review, that is, being referred to DOMEC.

We collected PGY-1 data on the students included in our study annually from Program Directors (PDs) who oversee the training of military medical trainees. All items in the evaluation form were to be rated on a 5-point Likert scale. For example, a PD would rate an intern’s “Ability to appreciate a patient’s illness in the context of their life” as “Unacceptable”, “Significantly below most PGY-1s”, “On par with most PGY-1s”, “Better than most PGY-1s”, or “Consistently at least one level higher than almost all PGY-1s”. Previous studies [5, 12] indicated that the items were loaded on two factors – Medical expertise and Professionalism. In this study, we used both of these factor scores. The response rate of PGY-1 PD evaluation form of this study cohort was 66.5% (we received 228 of 343 evaluation forms from PDs).

### Statistical analysis

As the students may have done multiple summaries, that is, the statements were evaluated more than one time by the raters, we first created four binary variables indicating whether or not a student received any of the four Unprofessionalism ratings (Unprofessional, Professional, Equivocal, and Unanswered). We then combined the ratings of Unprofessional and Equivocal and created a binary variable to indicate a student ever received either rating. We calculated the Pearson correlation coefficients to estimate the point-biserial correlation coefficients between this binary variable and other performance measures. In calculating the correlation coefficients for each pair of variables, the sample was those students who had data for both measures. The missing data were random. All the analyses were conducted in IBM SPSS Statistics version 22.

## Results

Listed below are exemplary comments coded in each category.

- Unprofessional – “*fat old dude with right leg swelling”; “I like pancakes. This guy has a DVT”.*
- Professional – “*12 y/o female presents with 2 days of runny nose, headache, productive cough, and reported high fevers. Associated symptoms include tiredness and achiness. On PE, tympanic membrane appears clear, with erythematous membranes on the back of her throat and cervical lymphadenopathy.”*
*“This is an obese man with progressive shortness of breath for the last six months and swelling of his legs for the last two weeks. No chest pain no orthopnea, no fever. He has expiatory wheezing on exam and has been coughing up sputum. He has a significant history of smoking and quit one month ago.”*
- Equivocal unprofessional (i.e., accurate, but too brief) – “*COPD”; “Obese”.*

Table 1 shows the frequency and percentage of the students who received each of the four ratings at least once – 68 (28.5%) for the category of Unprofessional, 183 (76.6%) for Professional, 42 (17.6%) for Equivocal, and 121 (50.6%) for Unanswered. Table 2 shows the descriptive statistics of the performance measures and the Pearson correlation coefficients between these measures and the combination of Unprofessional and Equivocal ratings. Statistically significant correlations existed between this combination and the Expertise score and the Professionalism score of PGY-1 PD evaluation. Receiving an Unprofessional or Equivocal rating was associated with lower Expertise score (*r* = −.19, *P* < .05) as well as lower Professionalism score (*r* = −.17, *P* < .05) during the first year of residency. The contingency table analysis indicated no significant association between the combined rating and referral to the student performance committee (*χ* ^2^ (1) = .01, *P* = .95). Unanswered (answers left blank) was not associated with adverse outcomes.

**Table 1:** Frequency and percentage of students who received the four ratings at least once (*N* = 343)

Rating	Number of students (percentage)
Unprofessional	68 (19.8%)
Professional	183 (53.4%)
Equivocal	42 (12.2%)
Unanswered	121 (35.3%)

**Table 2:** Pearson correlations between the combination of ratings of Unprofessional and Equivocal with other performance measures

	Medical school cumulative GPA	Step 1 score	Step 2 CK score	PGY-1 Expertise score	PGY-1 Professionalism score	DOMEC
	Mean (SD)					
Descriptive statistics	3.52 (.26)	219.39 (20.79)	230.54 (21.02)	3.79 (.78)	3.95 (.82)	11% got referred
Pearson correlations with the combination of ratings of Unprofessional and Equivocal	.07	−.01	.02	−.19*	−.17*	−.004

Note: In SPSS, Pearson correlation coefficient is calculated to estimate the point-biserial correlation coefficient between a binary variable and a continuous variable

*P < .05

## Discussion

According to a recent systematic report of U.S. and Canadian medical schools, incident reports and review of routine student evaluations are what most schools rely on to identify the majority of professionalism lapses [13]. But faculty and students are often reluctant to or unaware how to report. We have shown that review of brief student entries during MedU virtual patient cases may provide an additional, feasible marker for subsequent lower professionalism and performance ratings during internship. This echoes the future roles of virtual patients to apply educational-data-mining techniques to assess educational gains suggested by Berman et al. [8].

We tracked a cohort of students to determine if unprofessional or potentially unprofessional behavior-- as manifest in patient case summary statements during an ungraded, on-line assignment—weakly predicted subsequent poor performance in residency. Our study was unique in using such a low stakes clerkship measure for assessing unprofessional behavior and a group of longitudinally outcome measures with established reliability and validity.

Unprofessional behavior manifest in summary statements was correlated with residency performance measures, albeit with small effect. We believe that this makes intuitive sense in that program directors rate learners primarily on patient care measures in internship and the virtual patient cases are designed to be most like practice. Acts of omission (leaving an assignment blank) are arguably also unprofessional, but were not as associated with intern performance as were the deliberate entry of an unprofessional patient comment.

We found no associations between ungraded MedU professionalism coding and other graded elements in medical school. This may be because students give more effort to summative assessments than ungraded MedU assignments or that students are filtering their comments and behaviors on these more summative assessments. It could also be because only a subset of MedU cases are done (limited sampling, case specificity) whereas GPA and licensing exams are broader in scope. Alternatively, lack of association could suggest that virtual patient summary statement responses are reflecting something unique. Students likely perform differently during direct observation (clinical evaluations or OSCE examinations) then when on-line, alone, and presuming that there is no monitoring of their responses. While not part of this study, virtual patient cases and most computer-based exercises can also record time taken and time spent, which may be potential quantitative markers of student engagement, effort and organizational skills.

Our study has some limitations. It was a single-institution investigation, which limited the generalizability of the results. The statistically significant correlations found with this cohort were relatively weak. The study has several strengths. A formative, low-stakes, brief on-line assignment, the MedU virtual patient case summary text field response, was used as the screening tool. We believe this may be closer to social media posts or anonymous activities. This bypasses the Hawthorne effect of observation on behavior, reflecting what people do when they think no one is watching. This investigation is also a longitudinal follow up across both undergraduate medical education and graduate medical education outcome indicators. The PGY-1 program director evaluation form directly targets evaluation of professionalism in clinical settings.

## Conclusion

There was a significant association between unprofessionalism reflected by the students’ MedU summative comments and first year residency performance in both medical expertise and professionalism as judged by the program director. However, we did not find evidence of association between an unprofessionalism rating of student free text responses during a low stakes, on-line virtual patient case assignment and other medical school performance. Future studies can investigate whether the findings would change with larger, multi-institutional samples, and the added predictive value of including quantitative measures such as time of day or time spent on these computerized assignments.
